# Genomic associations with poxvirus across divergent island populations in Berthelot's pipit

**DOI:** 10.1111/mec.16461

**Published:** 2022-04-18

**Authors:** Eleanor C. Sheppard, Claudia A. Martin, Claire Armstrong, Catalina González‐Quevedo, Juan Carlos Illera, Alexander Suh, Lewis G. Spurgin, David S. Richardson

**Affiliations:** ^1^ 6106 School of Biological Sciences University of East Anglia Norfolk UK; ^2^ Grupo Ecología y Evolución de Vertebrados Instituto de Biología Facultad de Ciencias Exactas y Naturales Universidad de Antioquia Medellín Colombia; ^3^ Biodiversity Research Institute (CSIC‐Oviedo University‐Principality of Asturias) University of Oviedo Mieres Asturias Spain; ^4^ 8097 Department of Ecology and Genetics—Evolutionary Biology Evolutionary Biology Centre (EBC) Science for Life Laboratory Uppsala University Uppsala Sweden

**Keywords:** adaptation, avian pox virus, birds, genotype‐environment association, pathogen‐mediated selection

## Abstract

Understanding the mechanisms and genes that enable animal populations to adapt to pathogens is important from an evolutionary, health and conservation perspective. Berthelot's pipit (*Anthus berthelotii*) experiences extensive and consistent spatial heterogeneity in avian pox infection pressure across its range of island populations, thus providing an excellent system with which to examine how pathogen‐mediated selection drives spatial variation in immunogenetic diversity. Here, we test for evidence of genetic variation associated with avian pox at both an individual and population‐level. At the individual level, we find no evidence that variation in MHC class I and TLR4 (both known to be important in recognising viral infection) was associated with pox infection within two separate populations. However, using genotype‐environment association (Bayenv) in conjunction with genome‐wide (ddRAD‐seq) data, we detected strong associations between population‐level avian pox prevalence and allele frequencies of single nucleotide polymorphisms (SNPs) at a number of sites across the genome. These sites were located within genes involved in cellular stress signalling and immune responses, many of which have previously been associated with responses to viral infection in humans and other animals. Consequently, our analyses indicate that pathogen‐mediated selection may play a role in shaping genomic variation among relatively recently colonised island bird populations and highlight the utility of genotype‐environment associations for identifying candidate genes potentially involved in host‐pathogen interactions.

## 1 INTRODUCTION

Infection with pathogens can have considerable impact on individual fitness by reducing reproductive success, via increased mortality or morbidity (Anderson & May, 1979; Daszak et al., 2000). Consequently, pathogen‐mediated selection has the potential to affect the population dynamics, adaptation, and genetic variation of hosts (Hudson et al., 1998; O’Brien & Evermann, 1988; Ortego et al., 2007; Spurgin & Richardson, 2010). Selection on host immunity‐related genes—that is, those involved in pathogen recognition and elimination—may decrease within‐population genetic diversity and increase between‐population divergence as the alleles that provide the most benefit approach fixation (Mukherjee et al., 2009). However, evolutionary dynamics in natural disease systems are often more complex given that interactions between hosts and pathogens promote continual selection for reciprocal adaptations, resulting in an evolutionary arms race (Dawkins & Krebs, 1979; Paterson et al., 2010).

Selection as a result of diverse or rapidly evolving pathogens can promote and maintain genetic diversity in the host population (reviewed in Charlesworth, 2006; Spurgin & Richardson, 2010). Any selective regime that acts to maintain multiple alleles at a locus can be considered a mechanism of such “balancing” selection, including heterozygote advantage (Doherty & Zinkernagel, 1975), rare allele advantage (Slade & McCallum, 1992), and fluctuating selection (resulting from spatial and/or temporal changes in infection prevalence; Hill et al., 1991). However, these mechanisms are complicated and difficult to untangle in any given system, not least because they are nonexclusive, and may also interact with each other and with other processes (such as sexual selection) to shape genetic diversity (Apanius et al., 1997; Ejsmond et al., 2014). Understanding how pathogens and immune genes covary spatially within and across populations may provide some insight into the mechanisms and genes involved in adaptive evolution.

The search for pathogen associated genes has often involved a candidate gene approach (Bernatchez & Landry, 2003; Netea et al., 2012); however this is limited in its ability to include preidentified loci. Using genome‐wide markers would allow for discovery of further loci but even traditional population‐level based approaches may have limited power to detect small frequency shifts responsible for adaptation in polygenic traits (Pritchard et al., 2010). Alternatively, landscape genomic approaches such as genotype‐environment association studies, also called environmental association analyses, test for correlations between population patterns of allele frequencies and environmental factors across a spatial scale (Hoban et al., 2016; Rellstab et al., 2015). This method has been successfully employed, for example, to identify adaptation to the climatic environment in a range‐expanding damselfly (Dudaniec et al., 2018), to uncover parallel adaptive responses in congeneric grasshoppers along elevational gradients (Yadav et al., 2021), and to understand abiotic stress tolerance in barley landraces (Lei et al., 2019). Comparatively few genotype‐environment associations have attempted to identify genes that confer resistance to infectious diseases (but see Fraik et al., 2020; Mackinnon et al., 2016). We believe that such an approach could also overcome some of the difficulties that other individual‐based association methods experience when used for infectious disease data in wild populations. That is, rather than test for associations between individual genotypes and disease status—which could include individuals that have survived infection or are yet to be exposed to the pathogen in the same “no infection” set—testing for associations between allele frequencies and pathogen prevalence represents the selection generated by the pathogen in the population.

Avian pox is caused by a double‐stranded DNA virus (genus *Avipoxvirus*; family Poxviridae) and has been reported in numerous bird species worldwide (Bolte et al., 1999; van Riper & Forrester, 2007; Williams et al., 2021). Transmission occurs by biting insect vectors, skin‐to‐skin contact, or indirect contact when virions persist in the environment (van Riper et al., 2002; Smits et al., 2005). Infection manifests as proliferative lesions: the most common occur on featherless skin, but internal lesions can form on the diphtheritic membrane of the mouth, respiratory tract and digestive system (Tripathy, 1993). In severe cases, lesions may lead to impaired vision, feeding, flight and breathing, emaciation and death (Davidson et al., 1980; Docherty et al., 1991; Orós et al., 1997). Pox infections have been shown to have implications for host predation (Laiolo et al., 2007), secondary infections (van Riper & Forrester, 2007), male pairing success (Kleindorfer & Dudaniec, 2006), reproductive output (Lachish et al., 2012; Vanderwerf & Young, 2016) and productivity (Carrete et al., 2009). Severe population declines in endemic island species have even been linked to avian pox outbreaks (Alley et al., 2010; Kleindorfer & Dudaniec, 2006; van Riper et al., 2002). Although minor pox lesions typically heal, birds are often left with deformities, including missing digits and misshapen bills (van Riper et al., 2002; Vanderwerf, 2001), which may affect foraging success. Despite these fitness effects, few studies have investigated how pox infection varies both spatially and temporally (Carrete et al., 2009; Lawson et al., 2012; Samuel et al., 2018; Spurgin et al., 2012; Zylberberg et al., 2012), and none have investigated how it shapes patterns of host genetic diversity. Such information would provide insight into the selective pressure exerted by *Avipoxvirus* and its consequence for host immunogenetic adaptation in wild populations.

Berthelot's pipit (*Anthus berthelotii*)—a small passerine, endemic to three archipelagos of Macaronesia—provides an excellent natural system with which to investigate pathogen‐mediated selection and the evolution of immune genes. The pipit colonised the Canary Islands from Africa 2.5 Ma and from there colonised the Selvagens and the Madeiran archipelago in two independent events relatively recently c. 8500 years ago (Illera et al., 2007; Martin et al., 2021; Spurgin et al., 2014). Population bottlenecks associated with these colonisation events, and a lack of subsequent gene flow, have resulted in low genetic diversity within these populations and high genetic structuring across archipelagos (Armstrong et al., 2018; Illera et al., 2007; Martin et al., 2021; Spurgin et al., 2014). Importantly, pathogen pressures—specifically the prevalence of avian pox and malaria—are temporally consistent within populations but spatially variable between populations (Illera et al., 2008; Spurgin et al., 2012), and at a finer scale within populations (González‐Quevedo et al., 2014). Therefore, these pathogens are likely to have shaped host genetic diversity across populations of Berthelot's pipits.

Candidate loci that may be involved in combating avian pox infection have already been identified in Berthelot's pipit. At the population‐level, balancing selection appears to have maintained functionally divergent loci at the class I major histocompatibility complex (MHC) in Berthelot's pipit (González‐Quevedo et al., 2015a; Spurgin et al., 2011). The molecules encoded by these loci are key receptors involved in the acquired immune system presenting antigens in cells infected by intracellular pathogens, such as viruses (Hewitt, 2003). Similarly, while overall patterns of diversity at Toll‐like receptors (TLRs; involved in the innate immune response) are shaped by drift across the pipit's range, polymorphisms have been retained in most populations, and evidence for positive selection at some TLR loci exists (González‐Quevedo et al., 2015b). This includes TLR4, a locus that plays a role in virus sensing (Barton, 2007); TLR4‐dependent signalling has been shown to be important against influenza (Shinya et al., 2012) and vaccinia virus (the prototypic poxvirus; Hutchens et al., 2008). Evidence also exists for pathogen‐mediated selection on candidate immune genes within populations of Berthelot's pipits (Armstrong et al., 2019; González‐Quevedo et al., 2014, 2016); however, these studies have only focused on malaria as the selective agent. Thus, MHC class I and TLR4 are candidates for investigating immunogenetic adaptation to avian poxvirus. Nevertheless, these genes only represent a fraction of the 144 immune‐related genes identified in the avian genome (Ekblom et al., 2010) and it is also important to assess variation at a greater number of sites throughout the genome.

Here, we take advantage of the heterogeneity in pox prevalence within and between populations of Berthelot's pipit to identify loci that may be important in host response to pox infection. First, we test the hypothesis that variation at previously identified candidate immune effectors (TLR4 and MHC class I exon 3) will be associated with pox infection status in individual‐based analyses, consistent with pathogen‐mediated selection. We further hypothesise that differing pox‐mediated selective pressure among populations will shape the distribution of variation across the genome such that allele frequencies at the specific loci involved will show exceptional correlations with local pox prevalence. To test this, we perform a genotype‐environment association with genome‐wide restriction‐site associated DNA sequence (RAD‐seq) data from populations across the Berthelot's pipit range, encompassing the entire gradient of pox prevalence in this species. Finally, we identify likely candidate genes in close proximity to the loci identified in the genotype‐environment association, to assess the potential role they may play in adaptation to poxvirus.

## 2 MATERIALS AND METHODS

### 2.1 Field sampling and data collection

**FIGURE 1:**
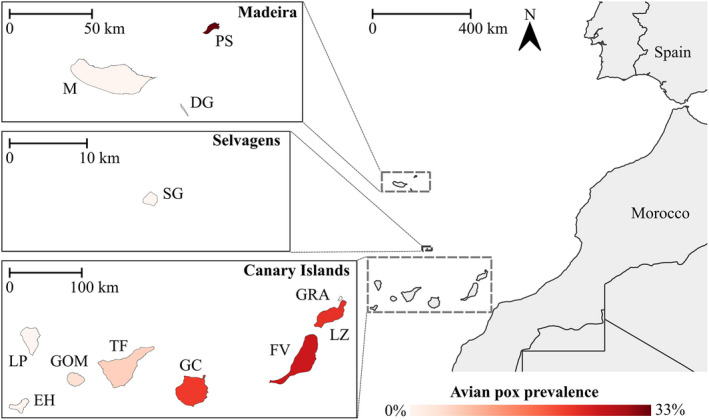
Map of the 12 islands sampled for Berthelot's pipits across its Macaronesian range. Populations are coloured according to their overall estimated pox prevalence across the 15‐year sampling period. M, Madeira; PS, Porto Santo; DG, Deserta Grande; SG, Selvagem Grande; LP, La Palma; EH, El Hierro; GOM, La Gomera; TF, Tenerife; GC, Gran Canaria; FV, Fuerteventura; GRA, La Graciosa; LZ, Lanzarote

Berthelot's pipits were caught during periods between 2005–2020 (Table S1) across 12 islands of their range (Figure 1). All sampling undertaken prior to 2019 has been described previously (Armstrong et al., 2019; González‐Quevedo et al., 2014; Illera et al., 2007; Spurgin et al., 2012). The remaining samples were collected in April 2019 (Lanzarote; *n* = 83) and February‐June 2020 (Lanzarote, La Graciosa, La Gomera and Tenerife; *n* = 200). Multiple sampling localities were chosen across each island to achieve a representative sample of the entire island population. In total, *n* = 1661 individuals were sampled.

Birds were captured in spring traps baited with *Tenebrio molitor* larvae. A blood sample (c. 25 μl) was taken by brachial venipuncture and stored in absolute ethanol (800 μl) at room temperature. Birds were ringed with a uniquely numbered metal band issued by the Spanish or Portuguese authorities as relevant. Age (adult/juvenile) was determined according to feather moult pattern (Cramp, 1985), and mass (±0.1 g), wing (±1 mm), tarsus, head length, and bill height, length and width (all ±0.1 mm) were measured.

To date, there is no reliable molecular/serological technique for diagnosing avian pox infection from blood samples (Baek et al., 2020; Farias et al., 2010; Smits et al., 2005; Williams et al., 2014). In our study, each bird was carefully assessed for evidence of pox infection—based on the presence/absence of lesions around the eyes, beak, feet, legs, or sparsely feathered areas. It is therefore possible that some infected birds may have been asymptomatic and incorrectly classified as uninfected. *Avipoxvirus* DNA has been amplified from samples taken from skin lesions for seven pipits included in this study (six from Porto Santo and one from Lanzarote), and identified as a single strain (see Illera et al., 2008). There is no evidence of other pathogens that result in similar lesions in this system. Previous studies have also identified poxvirus infection in Berthelot's pipit, and other species in the Canary Islands (Medina et al., 2004; Smits et al., 2005), including short‐toed larks (*Calandrella rufescens*) which inhabit the same shrub steppes. However, the specific strain of avian pox in Berthelot's pipits is not found in any of the other species, and thus appears to be host‐specific, potentially indicating long‐term coevolution between the strain and the host. Phylogenetic studies place this Berthelot's pipit lineage within the *Canarypox virus* clade, as an outlier to a subclade of lineages formed primarily of passerines (Gyuranecz et al., 2013; Illera et al., 2008).

DNA was extracted from blood following a salt extraction protocol (Richardson et al., 2001) and host sex was determined molecularly (Griffiths et al., 1998). To detect infection with *Haemoproteus* and *Plasmodium* spp. (here termed avian malaria for simplicity), we used a nested polymerase chain reaction (PCR) method described by Waldenström et al. (2004) to screen each sample at least twice (three times if the first two attempts gave contrasting results). Individuals were considered to be infected with malaria if the PCR produced a positive result twice, and all positive and negative controls gave expected results. For a subset of 400 infected individuals, the amplicon was sequenced to identify the strains present (see Armstrong et al., 2019; González‐Quevedo et al., 2014; Illera et al., 2008; Spurgin et al., 2012).

### 2.2 Sequencing and genotyping of candidate gene variants

To investigate genetic associations with pox at the individual level, we utilised targeted genotyping of TLR4 and MHC class I loci. The TLR4 data set had been generated previously for a study of associations with malaria (Armstrong et al., 2019); a complete description of methods and discussion of TLR4 variation can be found therein. In brief, 780 individuals were screened for TLR4 variation from the islands of Porto Santo (*n* = 190) and Tenerife (*n* = 590). These include individuals from a previous cross‐population study by González‐Quevedo et al. (2015b) in which 23–30 individuals were TLR4 genotyped from 13 Berthelot's pipit populations. A section of the extracellular region (660 bp) of the TLR4 gene (leucine‐rich repeat domain)—directly involved in pathogen recognition—was amplified using primers PauTLR4F and PauTLR4R (Grueber et al., 2012). The 129 samples from Porto Santo (2016) were genotyped using Sanger Sequencing, while the remaining individuals from Tenerife and Porto Santo (*n* = 577) were genotyped using KASP, a proprietary technology of LGC Genomics, as part of Armstrong et al. (2019). Five single nucleotide polymorphisms (SNPs) were reported, but one was later excluded because the SNP had a minor allele frequency (MAF) < 0.05 (Armstrong et al., 2019). Another of these SNPs was triallelic, therefore the least frequent alternate allele (T) was treated as missing (Porto Santo *n* = 1; Tenerife *n* = 12). Five nucleotide haplotypes were inferred using DnaSP version 6 (Librado & Rozas, 2009), and translated into four protein haplotypes (Armstrong et al., 2019; details regarding the SNPs, their positions, and the haplotypes are provided in Table S2). Samples with phase probabilities <0.90 were excluded from further analyses.

González‐Quevedo et al. (2015a) used 454 sequencing to screen variation at exon 3 of MHC class I (which partially encodes the antigen binding region) in 310 individuals from Tenerife (2011). In brief, two replicate PCR reactions were performed for each sample using different sets of fusion primers. Amplicon products were purified, pooled in equimolar amounts, and sequenced using a GS FLX Titanium system. Stringent variant/artefact identification and validation criteria were applied to identify putative MHC alleles (described in full in González‐Quevedo et al., 2015a) and 22 alleles were found within this population sample (GenBank accession numbers: JN799601‐JN799604, JN799606, JN799610‐JN799612, JN799623, JN799625, JN799636‐JN799639, JN799641, and KM593305‐KM593311). As recommended in González‐Quevedo et al. (2015a), we excluded two alleles with low amplification efficiencies (ANBE3 and ANBE31) from downstream analyses because their presence/absence cannot always be reliably ascertained for every individual, and one sample was discarded due to poor coverage. Here, the term “allele” refers to unique sequence variants amplified across a number of duplicated loci (the estimate for exon 3 of MHC class I genes in Berthelot's pipit is six loci; González‐Quevedo et al., 2015a).

### 2.3 RAD sequencing

To explore population‐level genetic associations with pox, we analysed previously published double digest RAD‐seq (ddRAD‐seq) data from 20 individuals selected from each population (22 from lowland Tenerife; Armstrong et al., 2018). Pipits on the mountain of El Teide (c. 2000 m above sea level) are considered a separate population from the rest of lowland Tenerife (Armstrong et al., 2018), thus there are 13 populations in this data set. Individuals screened were selected from the 2006 and 2009 sampling, in even sex ratios where possible, and across a wide geographical coverage within each population to reduce the likelihood of including related individuals.

Library preparation and initial bioinformatics followed the protocol in DaCosta and Sorenson (2014). Genotyping was performed by mapping reads to the zebra finch (*Taeniopygia guttata*) genome sequence (version 3.2.4; Warren et al., 2010). Within the “Berthelot's” data set (Armstrong et al., 2018)—used here for analyses—the loci that could not be confidently genotyped in a minimum of four samples, and those with missing or ambiguous genotypes for >10% of samples, were treated as missing data.

### 2.4 Identification of genetic variants associated with individual disease status

Generalised linear models (GLMs), with and without mixed effects, were used to identify predictors of pox infection at the individual level. For all models including genetic predictors, each island was modelled separately because population‐specific associations between genetic variation and pox may have evolved, and different genetic variants were present in the different populations (Armstrong et al., 2019). Predictor variables were assessed for collinearity using variance inflation factors (VIFs) or, in the case of categorical variables with more than two levels, generalised variance inflation factors (GVIFs; Fox & Monette, 1992). To obtain values equivalent to VIFs, GVIFs were transformed by squaring the standardised GVIF (GVIF^1/2df^, where df is degrees of freedom; Fox & Weisberg, 2019). VIFs ranged from 1.00–1.95 for all predictor variables used in the following models, thus none were excluded (threshold value: VIFs < 3; Zuur et al., 2010).

Prior to testing for associations with key candidate genes within populations, we used a multipopulation data set to model the relationship between nongenetic variables and pox infection status (not infected/infected) to build a base model using the maximum sample size. This data set consisted of all individuals screened for variation at candidate genes, but two individuals were excluded because the sexing PCR failed (*n* = 778). This generalised linear mixed model (GLMM) included age class (adult/juvenile), sex (male/female), island (Porto Santo/Tenerife) and malaria infection status (not infected/infected) as fixed factors, with sampling year as a random factor. We also considered interactions between age class and island, and malaria infection status and island. Nonsignificant predictors and interaction terms *(p* > .05) were removed sequentially leaving a minimal model (Bolker et al., 2009). Such an approach can inflate the probability of type 1 errors (Mundry & Nunn, 2009), thus all removed variables were re‐entered into the minimal model one at a time to determine their significance and parameter estimates. Genetic variables were later added to the minimal model to assess their significance in explaining variation in pox infection status.

GLMMs were used to test for associations between TLR4 variation and pox infection status. We focused on associations with protein haplotypes rather than single SNPs because these should better reflect functional differences at the TLR. Nonetheless, different protein haplotypes may have the same functional properties. In future, an in silico approach to assess the specific functional/structural/regulatory effects of these different variants, and ultimately their contribution to disease development, could be undertaken. Different TLR4 variants were represented as fixed factors in the following ways: (i) protein haplotype (presence/absence), (ii) protein haplotype heterozygosity (homozygote/heterozygote), and (iii) specific protein genotype (to investigate whether there is a synergistic effect when two specific protein haplotypes are present). Rare variants and genotypes (<0.05 in frequency) were removed from analyses (see Table S2) because they lack sufficient power to test effects. The effect of individual haplotypes, heterozygosity and genotypes were investigated separately to avoid problems of collinearity.

Associations between MHC class I variation and pox infection were assessed for birds sampled from Tenerife in 2011 (*n* = 309). We could not assign alleles to specific loci and resolve haplotypes because MHC alleles were amplified across multiple unidentified loci, thus we were unable to test for associations at the haplotype‐level. Instead, separate GLMs were performed to evaluate the effects of (i) MHC diversity (number of alleles per individual, 3–10) and optimality (the quadratic of allele number; to investigate whether intermediate MHC heterozygosity has the greatest fitness benefit), and (ii) individual MHC alleles (presence/absence). Rare and almost fixed MHC alleles (<0.05 and >0.95 in frequency) were removed from the second model because they provide no resolution. We also removed two alleles (ANBE16 and ANBE49) because they only occurred in individuals that were not infected with pox (12.00 and 7.44 in frequency, respectively) and therefore prevented model convergence. We detected a strong positive association between malaria and pox infection (see Section 3), and therefore considered the possibility that including malaria as a variable in the minimal model may have masked the genetic effects on pox infection status. Thus, all genetic models above were performed again without malaria infection status as a predictor.

For all models above, we fitted a binomial error structure and used a logit link function. All modelling was performed using R version 4.0.2 (R Development Core Team, 2020), with GLMMs constructed using the lme4 package (Bates et al., 2015), VIFs calculated using the car package (Fox & Weisberg, 2019), and the explained variance (R^2^) calculated according to the delta method (Nakagawa et al., 2017) using the r.squaredGLMM function in the MuMIn package (Barton, 2020).

### 2.5 Identification of SNPs correlated with population‐level pox prevalence

We applied a Bayesian approach to identify SNPs strongly associated with differences in pox prevalence across populations. This was implemented by Bayenv2.0, which first estimates a null model based on covariances of observed allele frequencies between populations that arise due to shared evolutionary history, and then assesses each SNP individually for linear correlations between population allele frequencies and environmental variables (Coop et al., 2010; Günther & Coop, 2013). We used the ddRAD data set, described above, and performed additional filtering steps in Plink version 1.9 (Chang et al., 2015) to generate a set of independent markers: a MAF threshold of 0.05 was applied to remove rare SNPs, and the remaining markers were filtered to remove loci in strong linkage disequilibrium (LD; Plink command: –indep‐pairwise 50 5 0.5). PGDspider (version 2.1.1.5; Lischer & Excoffier, 2012) was used to convert to a Bayenv input file format. Population covariance matrices were generated using this pruned marker set in ten replicate runs of Bayenv2.0, each of 100,000 iterations and with different seed numbers, to ensure convergence. The last matrices of the ten independent runs were averaged to obtain a final, single covariance matrix, and the equivalent correlation matrix (Table S3) was compared to previously published pairwise F_ST_ values derived from both microsatellites and the ddRAD‐seq SNPs (Illera et al., 2007; Martin et al., 2021) to ensure population structure was well estimated. In both cases, this matrix was consistent with the previous estimates of structure, indicating no problems in the labelling of populations.

To obtain best estimates of pox prevalence for each population and account for variability across different years due to natural fluctuations or sampling error, we used the complete field data set (2005–2020). Prevalence was calculated as the total number of individuals caught with visible pox lesions across all sampling years, divided by the total number of individuals caught across all sampling years. One bird from La Palma that was originally identified as having pox (Spurgin et al., 2012) was later revised after more experience diagnosing such infections, thus no confirmed cases of pox have been identified on La Palma. Prior to analyses with Bayenv2.0, the population prevalence estimates were standardised to a mean of zero and variance of one.

Using the mean covariance matrix estimated above as a null model, we ran Bayenv2.0 for five independent replicates, on the same pruned set of SNPs. We report estimates for both Bayes factor (BF) values (measure of support for the alternative model in which the genotype shows a linear association with the tested environmental variable compared to the null model) and non‐parametric Spearman's rank coefficients (ρ; strength of correlation between allele frequencies and the environmental variable). The latter served to reduce potential false positives as high BF values can also result from single outlying populations (Günther & Coop, 2013). Bayenv can also show high run‐to‐run variability (Blair et al., 2014). We sought to reduce false positives due to this variance by testing the concordance between BF values and Spearman's ρ across the five replicate runs at different numbers of iterations (100,000, 200,000, and 500,000 iterations) and averaging these estimates across runs as advised by Blair et al. (2014). At 500,000 iterations, the correlation observed between runs consistently reached >0.99 for Spearman's ρ and ranged from 0.08–0.53 for BF values. SNPs were therefore considered candidates if their average BF and average absolute value of Spearman's ρ across five replicate runs, each of 500,000 iterations, ranked in the highest 1% (BF ≥ 7.4) and 10%, respectively. BF values were interpreted according to a classification scheme adjusted from Jeffreys’ scale of evidence: BF > 3, BF > 10, BF > 30, BF > 100, indicative of moderate, strong, very strong and extreme evidence of selection, respectively (Lee & Wagenmakers, 2013; modified from Jeffreys, 1961). To check whether BF values of candidate SNPs were driven by the overall correlation across the pox prevalence gradient rather than a single population, we also visually investigated the allele frequency structure of candidate SNPs across populations: MAFs for all candidate SNPs were calculated within each population using Plink.

We compiled a list of candidate genes located within 10 kb up‐ or downstream of each candidate SNP in the zebra finch assembly Taeniopygia_guttata‐3.2.4 using NCBI Genome Data Viewer version 5.1 (www.ncbi.nlm.nih.gov/genome/gdv/browser). Additionally, we used BEDTools version 2.29.2 (Quinlan & Hall, 2010) and the genome positions of known immune‐related genes within the same zebra finch assembly, given by Ekblom et al. (2010), to quantify the proportion of avian immune genes that will have been overlooked for associations with pox prevalence when using our marker set and methods. Throughout, SNPs are identified by RAD‐tag ID, followed by distance in base pairs from the start of the tag.

## 3 RESULTS

### 3.1 Individual predictors of pox infection status

**FIGURE 2:**
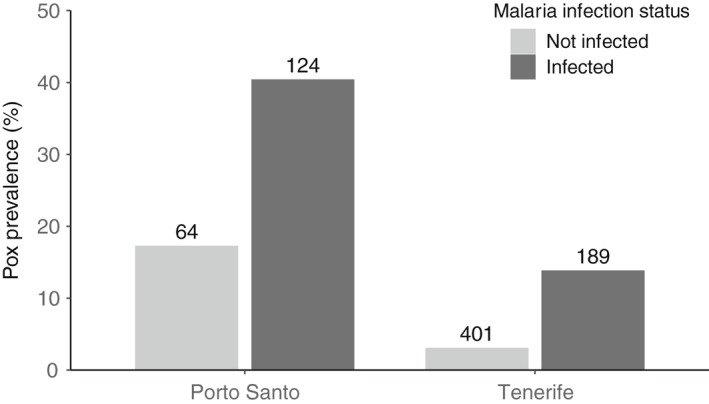
Prevalence of pox infection among Berthelot's pipits with and without malaria infection on Porto Santo and Tenerife. Numbers above the bars represent sample sizes

In Berthelot's pipits on Tenerife and Porto Santo, there was no significant association between age or sex and pox infection status (Table 1). However, there was a highly significant effect of malaria infection status and island identity (Table1 and Figure 2; marginal R^2^ = 0.16). Individuals infected with malaria had an increased likelihood of pox infection (i.e., 24.3%, compared to just 4.9% among individuals not infected with malaria) and pox prevalence was higher among individuals on Porto Santo (32.4%) than on Tenerife (6.4%). Thus, malaria infection status was retained as a predictor variable in subsequent genetic models.

**TABLE 1:** Generalised linear mixed models (GLMM) testing individual‐level predictors of pox infection status in Berthelot's pipits on Porto Santo and Tenerife (*n* = 778)

Fixed effects	Estimate	Std. error	Z	*p*‐value
Intercept	–1.678	0.342	–4.905	
Malaria	1.584	0.280	5.653	<.001***
Island identity	–1.682	0.362	–4.645	<.001***
Age	–0.123	0.406	–0.304	.761
Sex	0.180	0.296	0.608	.543

Random effects	Variance			
Sampling year	0.127	5 sampling years		

Note

Estimates and significance levels for each predictor represent the values upon re‐entry into the minimal model. Those in bold were retained in the minimal model. Reference categories for each predictor is as follows: malaria infection status = not infected, island identity = Porto Santo, sex = female, and age = adult. Significant terms: ***p < .001.

There was no association between TLR4 protein haplotype, heterozygosity, or genotype and pox infection status within individuals on either island when controlling for malaria infection status (Table 2), nor when malaria was removed from the models (Table S4).

**TABLE 2:** TLR4 variation in relation to pox infection status in Berthelot's pipits on Porto Santo and Tenerife

Porto Santo					Tenerife				
	Estimate	Std. error	z	*p*‐value		Estimate	Std. error	z	*p*‐value
Model (A)					Model (A)				
Fixed effects					Fixed effects				
Malaria	1.555	0.444	3.506	<.001***	Malaria	1.660	0.383	4.334	<.001***
TLR4_P1	–0.164	0.506	–0.324	.746	TLR4_P1	0.313	0.368	0.852	.394
TLR4_P2	–0.247	0.413	–0.598	.550	TLR4_P2	0.074	0.661	0.112	.911
TLR4_P3	–0.141	0.381	–0.369	.712					
Random effects	Variance				Random effects	Variance			
Sampling year	0.151	3 sampling years			Sampling year	0.096	4 sampling years		
*n* = 184					*n* = 578				
Model (B)					Model (B)				
Fixed effects					Fixed effects				
Malaria	1.545	0.443	3.486	<.001***	Malaria	1.655	0.383	4.327	<.001***
TLR4_het	0.040	0.348	0.116	.908	TLR4_het	0.279	0.353	0.790	.429
Random effects	Variance				Random effects	Variance			
Sampling year	0.151	3 sampling years			Sampling year	0.094	4 sampling years		
*n* = 184					*n* = 578				
Model (C)					Model (C)				
Fixed effects					Fixed effects				
Malaria	1.539	0.486	3.165	.002**	Malaria	1.660	0.383	4.334	<.001 ***
TLR4_genotype1,2	–0.187	0.511	–0.366	.714	TLR4_genotype1,2	0.074	0.661	0.112	.911
TLR4_ genotype1,3	–0.005	0.444	–0.011	.991	TLR4_genotype2,2	–0.239	0.663	–0.361	.718
TLR4_ genotype2,3	–0.054	0.577	–0.094	.925					
TLR4_ genotype3,3	–0.165	0.769	–0.215	.830					
Random effects	Variance				Random effects	Variance			
Sampling year	0.188	3 sampling years			Sampling year	0.096	4 sampling years		
*n* = 168					*n* = 578				

Note

Generalised linear mixed models (GLMMs) were used to test for associations between (A) TLR4 protein haplotype (presence/absence), (B) TLR4 protein haplotype heterozygosity (homozygote/heterozygote), (C) TLR4 protein genotype, and pox infection status. Reference factor levels: malaria infection status = not infected, TLR_P = absence, TLR4_het = homozygote, and TLR4_genotype = 1,1. Significant terms: **p < .01, and ***p < .001.

The GLM limited to individuals from Tenerife for which we had MHC variation information showed no association between MHC diversity, optimality, or individual alleles and pox infection status after controlling for malaria infection (Table 3) and when malaria was removed from the models (Table S5).

**TABLE 3:** Variation at MHC class I exon 3 in relation to pox infection status in Berthelot's pipits on Tenerife (*n* = 309)

Fixed effects	Estimate	Std. error	z	*p*‐value
Model (A)				
Malaria	1.787	0.570	3.138	.002**
N.alleles	–1.403	1.233	–1.138	.255
N.alleles.squared	0.100	0.093	1.082	.279
Model (B)				
Malaria	1.937	0.594	3.260	.001**
ANBE10	0.341	1.214	0.281	.779
ANBE8	–0.881	0.794	–1.109	.267
ANBE4	–0.059	0.572	–0.104	.917
ANBE43	–0.194	0.539	–0.360	.719
ANBE1	0.936	0.530	1.766	.077
ANBE44	0.548	0.765	0.716	.474
ANBE45	1.067	0.967	1.104	.270
ANBE9	1.029	0.680	1.513	.130
ANBE46	0.846	0.866	0.977	.329
ANBE47	–0.624	0.681	–0.917	.359
ANBE11	–0.408	0.705	–0.579	.563
ANBE6	–0.426	0.917	–0.465	.642
ANBE38	0.669	0.892	0.751	.453

Note

Generalised linear models (GLMs) were used to test for associations between (A) MHC diversity (number of alleles per individual, 3–10) and optimality (quadratic of MHC allele number), and (B) presence of specific MHC alleles (presence/absence), and pox infection status. Reference factor levels: malaria infection status = not infected, and ANBE = absence. Significant terms: **p < .01.

### 3.2 Signatures of pox‐driven selection at the population level

The ddRAD library produced by Armstrong et al. (2018) contained 9960 high‐quality SNPs. After filtering SNPs with a MAF of less than 0.05, we retained 3525. This data set was further reduced by removing SNPs in strong LD to generate a set of independent markers for analysis in Bayenv2.0, resulting in a final data set of 2334 SNPs.

Pox prevalence varied greatly among populations of Berthelot's pipit, ranging from 0% to 32.6% (shown in Table S6), but was broadly consistent within populations across the different sampling years (Figure S1). Population prevalence levels were highly correlated between 2006 and 2009 (2005 and 2009 for Selvagem Grande), when all populations had been sampled (Pearson correlation: *R* = 0.72, *p* = .005). We have never observed evidence of pox infection in the Selvagens, or in three islands of the Canaries (El Hierro, La Palma and La Graciosa) and two islands of the Madeiran archipelago (Deserta Grande and Madeira). Yet, the third island of the Madeiran archipelago, Porto Santo, had the highest pox prevalence of all populations. The remaining Canary Islands generally showed a negative east‐west gradient in pox prevalence (Figure 1), ranging from 25.6% to 5.4% (prevalence in the mountain population of El Teide was 1.9%).

**FIGURE 3:**
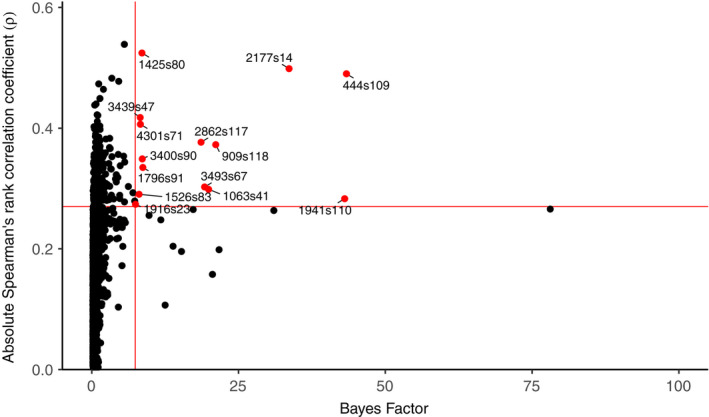
Bayes factor values versus absolute Spearman's rank correlation coefficients (ρ), averaged from five replicate runs, for genome‐wide ddRAD SNPs among 13 Berthelot's pipit populations. SNPs were considered candidates for adaptation to population‐level pox prevalence by Bayenv2.0 if they ranked in the highest 1% of Bayes factor values (≥7.4, threshold indicated by the vertical red line) and 10% of Spearman's ρ (threshold indicated by the horizontal red line). Fourteen SNPs were identified as candidates (those in red)

**FIGURE 4:**
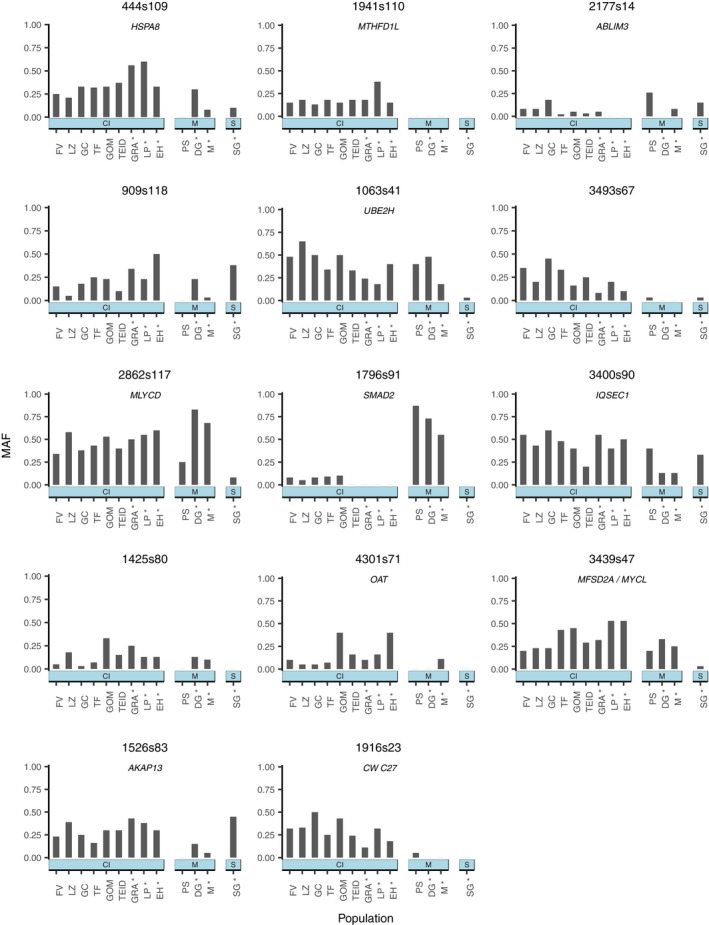
Minor allele frequency (MAF) distribution patterns of 14 candidate SNPs associated with population‐level pox prevalence identified by Bayenv2.0 across populations of Berthelot's pipit. Nearby genes are noted below the SNP names. Populations are first grouped by archipelago (CI, Canary Islands; M, Madeira; S, Selvagens) and then ordered according to population‐level pox prevalence (highest‐lowest). Pox‐free populations are indicated by an asterisk. FV, Fuerteventura; LZ, Lanzarote; GC, Gran Canaria; TF, Tenerife; GOM, La Gomera; TEID, Teide; GRA, La Graciosa; LP, La Palma; EH, El Hierro; PS, Porto Santo; DG, Deserta Grande; M, Madeira; SG, Selvagem Grande

Analysis with Bayenv2.0 identified 14 candidate SNPs (0.6% of total) where allele frequencies were highly associated with population‐level pox prevalence, as indicated by their BF value and Spearman's ρ (Figure 3) across five independent runs. Raw allele frequency patterns for these candidate SNPs showed variable trends across the gradient of pox prevalence (note that Bayenv2.0 accounts for patterns of demography in observed allele frequencies but the raw data is shown in Figure 4). Within the Canary Islands, the top candidate SNP (444s109) generally showed higher MAFs as pox prevalence decreased. The minor allele was also absent in Porto Santo only (Madeiran archipelago; highest overall pox prevalence). In contrast, some SNPs (e.g., 3493s67) showed lower MAFs as pox prevalence decreased. For other SNPs, such as 2177s14 and 1796s91, the minor allele was very rare or absent within populations free of pox in the Canary Islands, but generally had a higher prevalence within populations where pox had been recorded.

Of the 14 SNPs identified by Bayenv2.0, eight were located within annotated genes in the zebra finch genome (57.1%) identified using the NCBI genome browser. For three candidate SNPs, we identified genes within 10 kb up‐ or downstream, and there were three SNPs that were not close to genes (21.4%; closest genes 27,722–201,990 bp from SNP). In total, we identified 12 candidate genes from 11 SNPs (while SNP 3439s47 was located within the gene *MFSD2A*, it was also only approximately 4000 bp upstream of another gene, *MYCL*, so in this case both genes are identified). Ten of these identified genes have been implicated in cellular stress responses and/or have shown associations with viral infections (Table 4). Specific candidate SNPs that showed very strong evidence of selection (BF > 30) include those that were found approximately 6000 bp from the gene *HSPA8* (heat shock protein 70 family member 8), within the gene *MTHFD1L* (methylenetetrahydrofolate dehydrogenase NADP+dependent 1 like), and approximately 4000 bp from the gene *ABLIM3* (actin binding LIM protein family member 3). Of the immune genes identified by Ekblom et al. (2010) for which there was a mapped location, nine were located within 10 kb of a SNP typed in the present study, and were therefore assessed for an association with pox (c. 7%, Table S7).

**TABLE 4:** SNPs identified as associated with population‐level pox prevalence by Bayenv2.0 in Berthelot's pipits (those ranked in the highest 1% and 10% of Bayes factor [BF] values and Spearman's ρ respectively)

SNP	Genomic location (Chr: BP)	BF	ρ	Candidate gene(s)	Gene name/description	Putative function	Evidence for role in the response to viral infection
*444s109*	24:3526218	43.39	0.49	*HSPA8*(6319 bp US)	Heat shock protein family A (Hsp70) member 8	ATP‐dependent molecular chaperone that plays a role in protein folding processes	Hsp70 isoforms play a role in viral infection (Santoro et al., 2009), upregulated during poxvirus infection (Brum et al., 2003; Cheng et al., 2018; Jindal & Young, 1992; Kowalczyk et al., 2005; Sedger & Ruby, 1994).
*1941s110*	3:56769033	43.07	0.28	*MTHFD1L*(in gene)	Methylenetetrahydrofolate dehydrogenase (NADP + dependent) 1 like	Catalyses the synthesis of tetrahydrofolate in mitochondria in the folic acid cycle	Linked to avian influenza, may have regulatory role in replication (Zhang et al., 2020).
*2177s14*	13:1724905	33.60	0.50	*ABLIM3*(4111 bp US)	Actin binding LIM protein family member 3	Interacts with actin filaments and may occur within adherens junctions	Silencing reduced the replication of hepatitis C virus (Blackham et al., 2010).
*909s118*	21:5744237	21.12	0.37	—	—	—	—
*1063s41*	1A:217173	19.90	0.30	*UBE2H*(in gene)	Uubiquitin conjugating enzyme E2 H	Catalyses the attachment of ubiquitin to other proteins in the ubiquitin/proteasome degradation pathway	Linked to herpes simplex virus (Lutz et al., 2017) and identified in response to infection with Aleutian mink disease virus (Karimi et al., 2021).
*3493s67*	17:4549983	19.21	0.30	—	—	—	—
*2862s117*	11:1265660	18.60	0.38	*MLYCD*(3598 bp DS)	Malonyl‐CoA decarboxylase	Catalyses the conversion of malonyl‐CoA to acetyl‐CoA during fatty acid metabolism	Highly expressed in cells infected with influenza virus (Coombs et al., 2010; Dove et al., 2012; Kroeker et al., 2012).
*1796s91*	Z:421273	8.71	0.33	*SMAD2*(in gene)	SMAD family member 2	Transcriptional modulator and downstream effector of the transforming growth factor (TGF)‐β signalling pathway	Linked to West Nile virus (Slonchak et al., 2016), Epstein‐Barr virus (Wood et al., 2007), herpesvirus (Xu et al., 2011), and polyomavirus (Sung et al., 2014).
*3400s90*	12:1391473	8.60	0.35	*IQSEC1*(in gene)	IQ motif and sec7 domain ArfGEF 1	Guanine nucleotide exchange factor, involved in the regulation of ADP‐ribosylation factor (ARF) protein signal transduction	—
*1425s80*	9:25142202	8.55	0.52	—	—	—	—
*4301s71*	6:32665728	8.28	0.41	*OAT*(in gene)	Ornithine aminotransferase	Catalyses ornithine‐glutamate interconversion during metabolism of arginine and glutamine	Decreased expression in influenza infected cells (Ding et al., 2016) and herpes infected spat (Jouaux et al., 2013).
*3439s47*	23:3676275	8.26	0.42	*MFSD2A*(in gene) *MYCL*(4164 bp US)	Major facilitator superfamily domain containing 2A MYCL proto‐oncogene, bHLH transcription factor	Sodium‐dependent lysophosphatidylcholine transporter Transcription activity, regulating the expression of many proproliferative genes	Plays a role in the pathogenesis of Zika virus (Zhou et al., 2019). —
*1526s83*	10:14176084	8.05	0.29	*AKAP13*(in gene)	A‐kinase anchoring protein 13	Scaffold protein involved in assembling signalling complexes	Plays a role in the initiation of human immunodeficiency virus replication (König et al., 2008).
*1916s23*	Z:50640938	7.45	0.27	*CWC27*(in gene)	CWC27 spliceosome associated cyclophilin	Pre‐mRNA splicing factor recruited by the spliceosome	Many cyclophilins play a role in infection by diverse viruses (Frausto et al., 2013), including some poxviruses (Castro et al., 2003; Zhou et al., 2021).

Note

Candidate genes identified within ±10 kb windows upstream (US) or downstream (DS) of the focal SNP.

## 4 DISCUSSION

We used both a candidate gene approach and landscape genomic approach to identify variation associated with spatial heterogeneity in avian pox prevalence within and across island populations. Linear modelling of individual‐level infection data within two populations of Berthelot's pipit found no evidence that variation in two previously identified candidate genes (TLR4 and MHC class I) was associated with pox infection. However, we consistently observed a positive association between pox and malaria infection. At the population‐level across the species range using ddRAD‐seq data, we identified 14 sites across the genome that showed pox‐associated clinal patterns in allele frequency after controlling for population genetic structure. At these sites we identified 12 genes, many of which are involved in cellular stress response pathways and have been previously associated with infection by a range of viruses, including poxviruses, and in different animals, including humans (see Table 4).

Within‐species variation in pox infection has previously been attributed to ecological (van Riper et al., 2002; Samuel et al., 2018), anthropic (Carrete et al., 2009) and physiological factors (Zylberberg et al., 2012). Here, we assessed predictors of pox infection at the individual‐level using two populations of Berthelot's pipits so that we could control for these factors in later genetic models. We found no evidence for an effect of age or sex on pox infection status. Previous studies have shown little indication that sex influences pox infection prevalence (Ruiz‐Martínez et al., 2016; Samuel et al., 2018). Prevalence is commonly reported to be higher in juvenile birds than in adults (Buenestado et al., 2004; Gortázar et al., 2002; Ruiz‐Martínez et al., 2016), which authors have linked to immunological naivety. However, opposite patterns have also been observed (Atkinson et al., 2005; Smits et al., 2005).

Malaria infection was the most significant predictor of pox infection in our system. This result adds to a growing body of evidence that avian pox and malaria infections are not independent among individuals, but instead show positive associations, as previously documented in native Hawaiian birds (Atkinson et al., 2005; Atkinson & Samuel, 2010; Samuel et al., 2018) and Berthelot's pipits with a much smaller data set (Spurgin et al., 2012). Whether this association is due to simultaneous vector‐borne transmission, reduced immunity following infection by the first pathogen and therefore susceptibility to secondary infections, or differential mortality among individuals with singular and concomitant infections needs further investigation. It is possible the pipit host could contain variants at key genes that confer shared resistance/susceptibility to both pathogens. However, different taxa of pathogens (i.e., virus versus protist) are normally recognised by different receptors, for example, TLR3 specifically recognises viral DNA (Barton, 2007), though other components of the immune defences may be shared. In the present study, we did not detect any SNPs that had previously been identified as associated with malaria in Berthelot's pipits (Armstrong et al., 2018, 2019; González‐Quevedo et al., 2016; discussed further below). Alternatively, the association between avian pox and malaria might reflect increased likelihood of exposure to both pathogens. For example, artificial water sources and poultry farms were associated with increased local prevalence of malaria in the pipit system (González‐Quevedo et al., 2014), and in multiple birds species across Tenerife (Padilla et al., 2017), probably due to the increased local density of vectors and hosts at these sites. The same could be true for pox prevalence, but as of yet no landscape‐scale study has investigated the factors driving local pox prevalence in Berthelot's pipit. Though, similar effects of animal husbandry on poxvirus infection rates have been documented in the short‐toed lark in the Canary Islands (Carrete et al., 2009).

We examined whether host genetic variation could explain the observed variation in patterns of infection among individuals. Both TLR4 and MHC class I were considered potential candidates that may interact with poxvirus based on evidence of selection at these loci in Berthelot's pipit (González‐Quevedo et al., 2015a,b), and their involvement in the immune pathways associated with poxvirus pathogenesis (Guerin et al., 2002; Hutchens et al., 2008). Some variants included in this study were previously found to be associated with malaria infection and risk among pipits in Porto Santo and Tenerife (Armstrong et al., 2019; González‐Quevedo et al., 2016). However, after controlling for other predictors mentioned above, we did not find any evidence for an association between pox infection and TLR4 heterozygosity, or individual protein haplotypes or genotypes. Nor did we find evidence for a link with MHC diversity, optimality, or individual alleles as would be expected under different scenarios of heterozygote advantage, fluctuating selection or rare allele advantage, and optimized heterozygosity. Given we found such a strong association between pox and malaria infection, it is perhaps even more surprising we did not find that any candidate variants were associated with pox infection. However, some difficulty lies with the complexity of classifying individual infection status. For example, individuals with highly susceptible genotypes may be included in the set of noninfected individuals due to them never being exposed to *Avipoxvirus*. Also, most individuals we identified with pox are perhaps just those individuals that have successfully survived pox, rather than those particularly susceptible to pox. Both of these factors could obscure association data. Nevertheless, these loci may not play a direct role in pox infection. Unfortunately, we could not assess these genes for population‐level associations with pox prevalence because there were no markers close enough to these genes in our ddRAD data set. The closest SNP (SNP 3201s69) to the TLR4 gene was approximately 30 kb away. Passerine MHC genes are difficult to map (He et al., 2021); MHC class I genes have been identified on chromosomes 16 and 22 in zebra finch (Balakrishnan et al., 2010; Ekblom et al., 2011) but exact locations are unknown.

The candidate gene approach is also limited to key genes with an already hypothesised role in the host's response to a particular pathogen, thereby excluding the possibility of identifying new genes, especially those that participate in hitherto unknown mechanisms underlying host‐pathogen interactions. That said, the concentrated sampling effort, largely within the same population, focused on a single or few gene(s), tends to offer higher statistical power compared to genome‐wide approaches (Amos et al., 2011). Therefore, it is possible to detect population‐specific associations between genetic variants and infection with pathogens with such an approach (e.g., Bonneaud et al., 2006). Further, candidate gene approaches allow for thorough investigation of highly polymorphic loci, where we might expect alleles to differ across populations, while the genome‐wide population‐level Bayenv approach only used SNPs that are consistent across populations. Accordingly, the candidate gene approach is very useful, but may be more relevant once further candidates—potentially those involved in polygenic responses or understudied mechanisms—have been identified using genome scans or GWAS.

We identified novel candidate loci associated with population‐level avian pox prevalence using a ddRAD‐seq marker set. The highest‐ranking SNP identified (444s109) was located on chromosome 24, c. 6000 bp from the gene *HSPA8*. This gene encodes a member of the heat shock protein 70 (Hsp70) family—molecular chaperones that assist in protein folding, degradation, and trafficking (Kampinga & Craig, 2010; Mayer & Bukau, 2005). During viral infection, the cellular heat shock response is induced and Hsp70 genes are upregulated (e.g., Brum et al., 2003; Burch & Weller, 2004; Howe et al., 2016; Manzoor et al., 2014), however the role these genes serve remains unclear. Although Hsp70 proteins are essential for protecting cells from stress, stabilising the cell's own proteins, preventing viral replication (Li et al., 2011), and signalling to the innate immune system (Kim et al., 2013), they may also support viral genome replication (Manzoor et al., 2014; Ye et al., 2013) or be exploited as molecular chaperones to process or stabilise viral proteins (Taguwa et al., 2015; Zhang et al., 2015). Indeed, Hsp70 appears to play a role in poxvirus replication (Cheng et al., 2018; Jindal & Young, 1992) and in the suppression of apoptosis, which lengthens the time the poxvirus has for replication (Kowalczyk et al., 2005). Given the evidence above, *HSPA8* should be considered a strong candidate for involvement in interactions between hosts and avian poxvirus, but further work is needed to understand the mechanistic basis for how variation at this locus affects infection.

In addition to *HSPA8*, many of the genes linked to SNPs identified in our study are involved in enzymatic pathways and cell signalling transduction and have been linked to viral infection. Poxviruses, and other large viruses such as herpesvirus, have dedicated much of their genomes to encoding proteins that allow them to subvert antiviral mechanisms and regulatory controls (reviewed in Leão & Fonseca, 2014; McFadden & Murphy, 2000; Seet et al., 2003; Smith & Kotwal, 2002). These include proteins that mimic extracellular host immune molecules and block the innate immune response (Alcami, 2007; Alcami & Smith, 1992; Kotwal & Moss, 1988; Mann et al., 2008). Other proteins may mask signals between the infected cell and the acquired immune system, (Boshkov et al., 1992; Guerin et al., 2002), or interfere with intracellular pathways such as signalling from cytokines, the ubiquitin pathway (Zhang et al., 2009), and other processes that promote cell death (reviewed in Nichols et al., 2017). Among the avian pox‐associated loci in this study, we specifically found genes that are involved in ubiquitin pathways and cytokine signalling: *UBE2H*, which probably catalyses the modification of proteins for degradation (Stewart et al., 2016), and *SMAD2*, a downstream effector of the transforming growth factor (TGF)‐β signalling pathway, which regulates the transcription of target genes including those leading to apoptosis (Derynck et al., 1998). Interestingly, the genome of avian fowl pox virus contains a putative homologue of the eukaryotic TGF‐β gene, which is unique to this genera of poxviruses and is likely to have immunomodulatory effects (Afonso et al., 2000). Thus, our findings fit well with the known mechanisms of immune evasion employed by poxviruses and their interactions with host proteins.

Despite the strong association we observed between pox and malaria infection at the individual‐level, we could not control for malaria prevalence in the population‐level analysis. However, none of the 14 candidate SNPs identified in this study were located near (within 10 kb) the malaria‐associated SNPs previously identified using the same genome‐wide data set for Berthelot's pipit and an EigenGWAS analysis (Armstrong et al., 2018). Clearly further work is needed to explore the interacting effects of avian pox and malaria infection and to identify which specific candidate genes respond to either disease agent. This is an important question if we aim to understand the mechanisms and agents driving genetic variation in these genes.

When identifying candidate SNPs, we applied strict criteria to reduce false positive associations with pox prevalence. We acknowledge that this could also mean that other avian pox‐associated loci may have gone undetected. That our Bayenv analysis detected 14 candidate loci that were strongly (BF > 7.4) associated with avian pox prevalence after using the stringent cutoffs suggests that this pathogen may have influence on genetic variation within—and structuring among—populations of Berthelot's pipit. While RAD sequencing is a cost‐effective method for rapidly genotyping large numbers of polymorphisms, such reduced representation sequencing approaches are only able to assess a small portion of the genome for genotype‐environment associations. Indeed, we were only able to evaluate c. 7% of the previously identified avian immune‐related genes (Ekblom et al., 2010) for associations with pox prevalence. However, this is a consideration for any genome scan or genome‐wide association study (GWAS) that does not use high resolution whole‐genome sequencing. It does not undermine the validity of the associations that we do detect. The aim of this study was not to identify every possible correlation between allele frequencies and pox prevalence, but rather, to identify some strong candidate SNPs for future study of pathogen‐mediated selection in wild bird populations. Generally, the approach applied in this study should be considered just one of many complementary tools that can be used in the search for genes involved in host‐pathogen interactions; however, it is by no means exhaustive.

Further studies in Berthelot's pipits and/or other pox‐infected avian populations are needed to validate the candidate loci identified in the current study and to identify other loci under selection from avian pox. In Berthelot's pipit, spatial variation in pox prevalence is independent of neutral population structure across the range, but is shaped by certain biogeographical factors (Spurgin et al., 2012), that is, smaller and more isolated islands are less likely to be infected with pox. This suggests that biogeographical factors, and other environmental factors, largely determine patterns of pox prevalence across populations. The Bayenv analysis is specifically designed to control for neutral population structure. Therefore, it is unlikely that the associations we observed between avian pox prevalence and specific SNPs were due to the prior distribution of susceptibility alleles caused by demographic history; rather associations are likely to be a consequence of pathogen‐mediated selection pressure. Nevertheless, studies assessing the infection status, health and survival of a large number of genotyped individuals within pox‐infected populations are necessary to verify the role of the candidate loci in pox resistance. Ultimately, these tests provide evidence of an association, not necessarily a causal link and thus, an empirical demonstration of their function is required, either using gene expression profiling among infected and noninfected individuals or a direct assay (Pardo‐Diaz et al., 2015). We acknowledge this is more difficult for a non‐model organism and endemic species such as Berthelot's pipit. Testing for signatures of selection using resequencing of candidate SNPs in individual birds would also corroborate whether selection is acting on specific genes. In addition, resequencing candidates across temporal samples either across generations in contemporary populations (e.g., Davies et al., 2021) or over longer time spans using museum samples (e.g., Alves et al., 2019) could enable inference of selection and may help to elucidate the mechanisms of adaptation by testing for allele frequency changes at these loci (Bank et al., 2014; Malaspinas, 2016).

## 5 CONCLUSIONS

Our study is one of the first to attempt to identify loci involved in avian poxvirus response across wild passerine populations. Using two different approaches, we were able to test for associations between disease and host genetic variation at both an individual and population level. Genotype‐environment associations were detected across populations of Berthelot's pipits exposed to different pox prevalence levels after controlling for demographic history/neutral genetic population structure. Thus, the results potentially evidence the evolution of hosts in in response to local pathogen pressure, but this needs to be confirmed in the future using extensive within‐population level analyses to link variants to the presence/absence of pox and/or individual survival. We identified novel pox‐associated genes involved in cellular stress signalling and immune responses, such as the heat shock response, cytokine signalling pathways, and apoptosis. We suggest that these genes represent promising new candidates with which to understand pathogen‐mediated selection in wild bird populations.

## AUTHOR CONTRIBUTION

Eleanor C. Sheppard and David S. Richardson designed the study, with input from Lewis G. Spurgin and Claudia A. Martin. Fieldwork was undertaken by Eleanor C. Sheppard, Claudia A. Martin, Claire Armstron, Catalina González‐Quevedo, Juan Carlos Illera, Lewis G. Spurgin and David S. Richardson. Claire Armstrong and Catalina González‐Quevedo developed the genetic data sets. Eleanor C. Sheppard performed data analysis and drafted the manuscript with supervision from David S. Richardson, Lewis G. Spurgin and Alexander Suh. All authors provided critical contributions to this study and approved the submission of this manuscript.

## CONFLICT OF INTEREST

The authors declare that they have no conflict of interest.

## Supporting information

Supplementary Material

## Data Availability

All data and the code used to perform the data analysis within this article are openly available in the Dryad Digital Repository: https://doi.org/10.5061/dryad.6t1g1jx0r.
