# Supplementary material for: Genomic associations with poxvirus across divergent island populations in Berthelot's pipit
Source: Mol Ecol. 2022 Apr 18;31(11):3154–73. doi: 10.1111/mec.16461 (PMC9321574; doi:10.1111/mec.16461)
Supplement: Supplementary file 1 — Supplementary Material [file MEC-31-3154-s001.docx]

**Supplemental Information for:**

**Genomic associations with poxvirus across divergent island populations in Berthelot’s pipit**

Eleanor C. Sheppard, Claudia A. Martin, Claire Armstrong, Catalina González-Quevedo, Juan Carlos Illera, Alexander Suh, Lewis G. Spurgin & David S. Richardson

**Table S1.** Sampling periods for the 13 populations of Berthelot’s pipit across Macaronesia and their use in different datasets in this study. Where the number of individuals included in each dataset is less than the total sample size for that sampling period, the number of individuals is denoted in brackets.

| **Archipelago** | **Population** | Year | Month/s | *n* Samples | Dataset/s |  |  |  |
| --- | --- | --- | --- | --- | --- | --- | --- | --- |
|  |  |  |  |  | Pox prevalence | TLR4 variation | MHC I variation | ddRAD-seq |
| *Madeira* | Deserta Grande | 2006 | Sept-Oct | 31 | X |  |  | X (18) |
|  |  | 2009 | Apr, Sept-Oct | 4 | X |  |  | X (2) |
|  | Madeira | 2005 | Apr | 1 | X |  |  |  |
|  |  | 2006 | Sept | 32 | X |  |  |  |
|  |  | 2009 | Mar-Apr, Sept-Oct | 29 | X |  |  | X (20) |
|  |  | 2016 | Apr-May | 29 | X |  |  |  |
|  | Porto Santo | 2006 | Sept | 31 | X | X |  | X (3) |
|  |  | 2009 | Mar | 30 | X | X |  | X (17) |
|  |  | 2016 | May-June | 129 | X | X |  |  |
| *Selvagens* | Selvagem Grande | 2005 | Apr | 52 | X |  |  |  |
|  |  | 2009 | Mar | 42 | X |  |  | X (20) |
| *Canary Islands* | La Graciosa | 2006 | Mar | 24 | X |  |  | X (2) |
|  |  | 2009 | Jan | 26 | X |  |  | X (18) |
|  |  | 2020 | Mar | 44 | X |  |  |  |
|  | Lanzarote | 2006 | Mar | 13 | X |  |  |  |
|  |  | 2008 | Nov | 1 | X |  |  |  |
|  |  | 2009 | Jan | 30 | X |  |  | X (20) |
|  |  | 2019 | Apr | 83 | X |  |  |  |
|  |  | 2020 | Feb-Apr | 113 | X |  |  |  |
|  | Fuerteventura | 2006 | Feb-Mar | 12 | X |  |  | X (2) |
|  |  | 2008 | Nov | 1 | X |  |  |  |
|  |  | 2009 | Jan | 30 | X |  |  | X (18) |
|  | Gran Canaria | 2006 | Jan-Feb | 31 | X |  |  |  |
|  |  | 2009 | Jan-Feb | 33 | X |  |  | X (20) |
|  | El Teide | 2006 | Mar-Apr | 30 | X | X (29) |  |  |
|  |  | 2008 | Oct | 3 | X |  |  |  |
|  |  | 2009 | Apr-May, Aug | 22 | X | X |  | X (20) |
|  |  | 2011 | Mar-Apr | 30 | X | X | X (26) |  |
|  |  | 2020 | May-June | 19 | X |  |  |  |
|  | Tenerife | 2005 | May | 7 | X |  |  |  |
|  |  | 2006 | Feb-Mar | 25 | X | X (22) |  | X (8) |
|  |  | 2009 | Jan-Aug | 35 | X | X (34) |  | X (14) |
|  |  | 2010 | Apr-May | 96 | X | X |  |  |
|  |  | 2011 | Jan-May | 358 | X | X (357) | X (284) |  |
|  |  | 2020 | May-June | 18 | X |  |  |  |
|  | La Gomera | 2006 | Feb | 30 | X |  |  | X (7) |
|  |  | 2009 | Jan, Apr | 20 | X |  |  | X (13) |
|  |  | 2020 | June | 6 | X |  |  |  |
|  | La Palma | 2006 | Jan-Feb | 28 | X |  |  | X (3) |
|  |  | 2009 | Feb, July | 22 | X |  |  | X (17) |
|  | El Hierro | 2006 | Jan-Feb | 31 | X |  |  |  |
|  |  | 2009 | Feb | 30 | X |  |  | X (20) |

**Table S2.** Details of TLR4 variation among Berthelot’s pipits on Porto Santo (PS) and Tenerife (TF) used in the current study.

| **Nucleotide haplotype** | Nucleotide sequence | | | | **Protein haplotype** | Amino acid sequence | Protein haplotype frequency^[[1]](#footnote-1)^ | |
| --- | --- | --- | --- | --- | --- | --- | --- | --- |
|  | SNP1 | SNP2 | SNP3 | SNP4 ^[[2]](#footnote-2)^ |  |  |  |  |
|  | (905 bp) | (970 bp) | (990 bp) | (992 bp) |  |  | PS | TF |
| *1* | A | G | T | A | *TLR4_P1* | DGPK | 0.53 | 0.29 |
| *2* | A | G | C | C | *TLR4_P2* | DGPT | 0.17 | 0.71 |
| *3* | A | G | T | C | *TLR4_P2* | DGPT |  |  |
| *4* | A | A | C | C | *TLR4_P3* | DDPT | 0.26 | 0.00 |
| *5* | G | G | C | C | *TLR4_P4* | GGPT | 0.04 | 0.00 |

**Table S3.** Equivalent correlation matrix to the final population covariance matrix (averaged from 10 independent runs) which was used as a null model in Bayenv2.0 analyses. Acronyms: GRA = La Graciosa; LZ = Lanzarote; FV = Fuerteventura; GC = Gran Canaria; TF = Tenerife; TEID = Teide; GOM = La Gomera; LP = La Palma; EH = El Hierro; SG = Selvagem Grande; M = Madeira; PS = Porto Santo; DG = Deserta Grande.

|  | GRA | LZ | FV | GC | TF | TEID | GOM | LP | EH | SG | M | PS | DG |
| --- | --- | --- | --- | --- | --- | --- | --- | --- | --- | --- | --- | --- | --- |
| GRA |  |  |  |  |  |  |  |  |  |  |  |  |  |
| LZ | 0.955 |  |  |  |  |  |  |  |  |  |  |  |  |
| FV | 0.955 | 0.958 |  |  |  |  |  |  |  |  |  |  |  |
| GC | 0.954 | 0.957 | 0.959 |  |  |  |  |  |  |  |  |  |  |
| TF | 0.953 | 0.958 | 0.959 | 0.959 |  |  |  |  |  |  |  |  |  |
| TEID | 0.953 | 0.958 | 0.959 | 0.959 | 0.960 |  |  |  |  |  |  |  |  |
| GOM | 0.950 | 0.954 | 0.956 | 0.956 | 0.956 | 0.956 |  |  |  |  |  |  |  |
| LP | 0.952 | 0.956 | 0.958 | 0.958 | 0.957 | 0.957 | 0.956 |  |  |  |  |  |  |
| EH | 0.946 | 0.950 | 0.952 | 0.952 | 0.952 | 0.953 | 0.953 | 0.952 |  |  |  |  |  |
| SG | 0.742 | 0.745 | 0.747 | 0.747 | 0.745 | 0.751 | 0.743 | 0.741 | 0.748 |  |  |  |  |
| M | 0.661 | 0.680 | 0.677 | 0.684 | 0.682 | 0.686 | 0.681 | 0.674 | 0.668 | 0.497 |  |  |  |
| PS | 0.650 | 0.670 | 0.666 | 0.673 | 0.671 | 0.675 | 0.670 | 0.662 | 0.657 | 0.482 | 0.975 |  |  |
| DG | 0.655 | 0.674 | 0.671 | 0.679 | 0.677 | 0.681 | 0.677 | 0.67 | 0.663 | 0.493 | 0.961 | 0.962 |  |

**Table S4**. Results of generalised linear mixed models used to investigate associations between TLR4 variation and pox infection status when the variable malaria infection status was removed: (A) TLR4 protein haplotype (presence/absence), (B) TLR4 protein haplotype heterozygosity (homozygote/heterozygote), (C) TLR4 protein genotype. Reference factor levels: TLR4_P = absence, TLR4_het = homozygote, and TLR4_genotype = 1,1.

|  | **Porto Santo** | | | | | **Tenerife** | | | | |
| --- | --- | --- | --- | --- | --- | --- | --- | --- | --- | --- |
| **Model (A)** | **Fixed effects** | Estimate | Std. Error | z | *P*-value | **Fixed effects** | Estimate | Std. Error | z | *P*-value |
|  | *TLR4_P1* | -0.145 | 0.476 | -0.305 | 0.761 | *TLR4_P1* | 0.222 | 0.360 | 0.616 | 0.538 |
|  | *TLR4_P2* | -0.203 | 0.392 | -0.517 | 0.605 | *TLR4_P2* | 0.085 | 0.647 | 0.131 | 0.895 |
|  | *TLR4_P3* | -0.093 | 0.363 | -0.256 | 0.798 |  |  |  |  |  |
|  | **Random effects** | Variance | Std. Dev. |  | | **Random effects** | Variance | Std. Dev. |  | |
|  | *Sampling year* | 4.676e^-15^ | 6.838e^-08^ | 3 sampling years | | *Sampling year* | 4.000e^-14^ | 2.000e^-07^ | 4 sampling years | |
|  | ***n* = 184** |  |  |  | | ***n* = 578** |  |  |  | |
| **Model (B)** | **Fixed effects** | Estimate | Std. Error | z | *P*-value | **Fixed effects** | Estimate | Std. Error | z | *P*-value |
|  | *TLR4_het* | 0.097 | 0.332 | 0.292 | 0.770 | *TLR4_het* | 0.201 | 0.345 | 0.583 | 0.560 |
|  | **Random effects** | Variance | Std. Dev. |  | | **Random effects** | Variance | Std. Dev. |  | |
|  | *Sampling year* | 0 | 0 | 3 sampling years | | *Sampling year* | 4.000e^-14^ | 2.000e^-07^ | 4 sampling years | |
|  | ***n* = 184** |  |  |  | | ***n* = 578** |  |  |  | |
| **Model (C)** | **Fixed effects** | Estimate | Std. Error | z | *P*-value | **Fixed effects** | Estimate | Std. Error | z | *P*-value |
|  | *TLR4_genotype1,2* | -0.152 | 0.484 | -0.313 | 0.754 | *TLR4_genotype1,2* | 0.085 | 0.648 | 0.131 | 0.895 |
|  | *TLR4_ genotype1,3* | 0.059 | 0.421 | 0.140 | 0.889 | *TLR4_genotype2,2* | -0.137 | 0.650 | -0.210 | 0.834 |
|  | *TLR4_ genotype2,3* | 0.031 | 0.554 | 0.056 | 0.956 |  |  |  |  |  |
|  | *TLR4_ genotype3,3* | -0.257 | 0.742 | -0.346 | 0.729 |  |  |  |  |  |
|  | **Random effects** | Variance | Std. Dev. |  | | **Random effects** | Variance | Std. Dev. |  | |
|  | *Sampling year* | 0 | 0 | 3 sampling years | | *Sampling year* | 0 | 0 | 4 sampling years | |
|  | ***n* = 168** |  |  |  | | ***n* = 578** |  |  |  | |

**Table S5.** Results of generalised linear models used to investigate associations between MHC class I variation and pox infection status when the variable malaria infection status was removed: (A) MHC diversity (number of alleles per individual, 3-10) and optimality (quadratic of MHC allele number), and (B) presence of specific MHC alleles (presence/absence). Reference factor levels: ANBE = absence.

|  | **Fixed effects** | Estimate | Std. Error | z | P-value |
| --- | --- | --- | --- | --- | --- |
| **Model (A)** | *N.alleles* | -1.530 | 1.191 | -1.284 | 0.199 |
|  | *N.alleles^2^* | 0.111 | 0.090 | 1.234 | 0.217 |
| **Model (B)** | *ANBE10* | 0.518 | 1.160 | 0.447 | 0.655 |
|  | *ANBE8* | -0.819 | 0.731 | -1.120 | 0.263 |
|  | *ANBE4* | -0.082 | 0.543 | -0.152 | 0.879 |
|  | *ANBE43* | -0.217 | 0.525 | -0.413 | 0.680 |
|  | *ANBE1* | 0.798 | 0.509 | 1.568 | 0.117 |
|  | *ANBE44* | 0.382 | 0.702 | 0.545 | 0.586 |
|  | *ANBE45* | 1.150 | 0.900 | 1.278 | 0.201 |
|  | *ANBE9* | 0.874 | 0.653 | 1.340 | 0.180 |
|  | *ANBE46* | 0.616 | 0.833 | 0.739 | 0.460 |
|  | *ANBE47* | -0.646 | 0.667 | -0.968 | 0.333 |
|  | *ANBE11* | -0.186 | 0.682 | -0.272 | 0.785 |
|  | *ANBE6* | -0.035 | 0.851 | -0.041 | 0.967 |
|  | *ANBE38* | 0.634 | 0.837 | 0.757 | 0.449 |

**Table S6.** Prevalence of avian pox in 13 populations of Berthelot’s pipit across Macaronesia. Prevalence was estimated from field data collected from 2005-2020 (the number of individuals caught with characteristic pox lesions divided by the total number of individuals caught throughout the entire period). The prevalence in each island was standardised by subtracting the mean and dividing by the standard deviation of prevalence across populations. Standardised prevalence was then used as an input variable for environmental association analyses.

| **Archipelago** | **Population** | Sample size | Pox prevalence % | Standardised prevalence |
| --- | --- | --- | --- | --- |
| *Madeira* | Deserta Grande | 35 | 0.00 | -0.75 |
|  | Madeira | 91 | 0.00 | -0.75 |
|  | Porto Santo | 190 | 32.63 | 1.95 |
| *Selvagens* | Selvagem Grande | 94 | 0.00 | -0.75 |
| *Canary Islands* | La Graciosa | 94 | 0.00 | -0.75 |
|  | Lanzarote | 240 | 23.33 | 1.18 |
|  | Fuerteventura | 43 | 25.58 | 1.36 |
|  | Gran Canaria | 64 | 21.88 | 1.06 |
|  | El Teide | 104 | 1.92 | -0.59 |
|  | Tenerife | 539 | 7.24 | -0.15 |
|  | La Gomera | 56 | 5.36 | -0.31 |
|  | La Palma | 50 | 0.00 | -0.75 |
|  | El Hierro | 61 | 0.00 | -0.75 |

**Table S7.** Detectable immune genes and their overlapping SNPs in the ddRAD-seq dataset for analyses with Bayenv. Genes and their genomic locations taken from Ekblom et al. (2010).

| **Avian immune gene** | Chromosome | Gene start position | Gene end position | SNP | SNP position |
| --- | --- | --- | --- | --- | --- |
| CD247 | 1 | 102472923 | 102479033 | 2003s115 | 102472695 |
| CD200 | 1 | 114120531 | 114124170 | 3088s53 | 114125011 |
|  |  |  |  | 3088s65 | 114125023 |
| TNFRSF11B | 2 | 142879487 | 142894631 | 2752s29 | 142880794 |
| CTSB | 3 | 110883194 | 110889580 | 2361s40 | 110887301 |
| Gal 8 | 3 | 110809584 | 110810285 | 397s64 | 110810106 |
|  |  |  |  | 397s76 | 110810118 |
| CD81 | 5 | 13447673 | 13455198 | 2998s79 | 13440274 |
| CD28 | 7 | 50658 | 60186 | 2150s95 | 51599 |
|  |  |  |  | 2150s39 | 51655 |
|  |  |  |  | 2150s37 | 51657 |
|  |  |  |  | 2150s15 | 51679 |
| TNFSF15 | 17 | 2727000 | 2741576 | 2140s105 | 2727606 |
| SOCS3 | 18 | 1664631 | 1665260 | 3441s90 | 1660387 |

**Figure S1.** Dot plot showing multiple pox prevalence values from different years within Berthelot’s pipit populations. Values from the same population tend to be similar. All populations have been sampled at least twice. Prevalence values only included where *n* > 10. FV = Fuerteventura; LZ = Lanzarote; GC = Gran Canaria; TF = Tenerife; GOM = La Gomera; TEID = Teide; GRA = La Graciosa; LP = La Palma; EH = El Hierro; PS = Porto Santo; DG = Deserta Grande; M = Madeira; SG = Selvagem Grande.

1. Positions based on the TLR4 protein coding region in the zebra finch genome.

   A fifth SNP (1010 bp, nonsynonymous) was excluded due to low minor allele frequency.

   Low frequency variants (< 0.05) were excluded from analyses. [↑](#footnote-ref-1)
2. This tri-allelic SNP was treated as biallelic by excluding the least frequent allele (T). [↑](#footnote-ref-2)
